# Propeller Flaps: A Review of Indications, Technique, and Results

**DOI:** 10.1155/2014/986829

**Published:** 2014-05-26

**Authors:** Salvatore D'Arpa, Francesca Toia, Roberto Pirrello, Francesco Moschella, Adriana Cordova

**Affiliations:** Chirurgia Plastica e Ricostruttiva, Dipartimento di Discipline Chirurgiche, Oncologiche e Stomatologiche, Università degli Studi di Palermo, Via del Vespro, 129 90127 Palermo, Italy

## Abstract

In the last years, propeller flaps have become an appealing option for coverage of a large range of defects. Besides having a more reliable vascular pedicle than traditional flap, propeller flaps allow for great freedom in design and for wide mobilization that extend the possibility of reconstructing difficult wounds with local tissues and minimal donor-site morbidity. They also allow one-stage reconstruction of defects that usually require multiple procedures. Harvesting of a propeller flap requires accurate patient selection, preoperative planning, and dissection technique. Complication rate can be kept low, provided that potential problems are prevented, promptly recognized, and adequately treated. This paper reviews current knowledge on propeller flaps. Definition, classification, and indications in the different body regions are discussed based on a review of the literature and on the authors' experience. Details about surgical technique are provided, together with tips to avoid and manage complications.

## 1. Introduction

The term “propeller flap” was first used in 1991 by Hyakusoku et al. [1] to describe an adipocutaneous flap based on a central subcutaneous pedicle, with a shape resembling a propeller that was rotated 90 degrees.

In 2006, combining the concept of propeller flaps and perforator based flaps, Hallock [2] reported a fasciocutaneous flap that was similar in shape to the one described by Hyakusoku but was based on a skeletonized perforating vessel and was rotated 180 degrees on an eccentric pivot point. Teo [3] gave the greatest contribution to the surgical technique and the application of the perforator propeller flap.

In the last years, the introduction of the propeller flaps gained great popularity; these flaps have been increasingly used for reconstruction of soft tissue defects of different parts of the body, and surgical technique has been refined and well described by several authors [4–9]. Perforator propeller flaps have a reliable vascular pedicle and can undergo wide mobilization and rotation; their harvest is fast and easy and does not require microsurgery; however, accurate patient selection, preoperative planning, and dissection technique are mandatory to prevent complications.

In this paper, recommendations for judicious planning of perforator propeller flaps in different body areas are provided; technical refinements and tips on how to avoid common mistakes are discussed.

## 2. Materials and Methods

Pertinent literature was collected: a pubmed search was performed using the keywords propeller flap, perforator flap, and freestyle flap. Forty papers were eventually included in this review. Definition, classification, and indications of propeller flaps in the different body regions are discussed; details about surgical technique are provided, together with tips to avoid and manage complications, based on selected relevant articles and on the authors' experience.

## 3. Results and Discussion

Propeller flaps can either be based on a known source vessel or can be harvested from any anatomical region as long as a Doppler signal of a perforator is detected (free-style propeller flaps).

The advantages of perforator propeller flaps can be summarized as follows [3, 4, 6, 8].

- They allow for a great freedom in design and choice of the donor site, based on the quality and volume of soft tissue required and on scar orientation.
- They represent a simpler and faster alternative to free flaps and expand the possibilities of reconstructing difficult wounds with local tissues.
- Their harvest is easy and fast, provided that appropriate dissection technique is applied.
- Donor site morbidity is kept very low, avoiding the sacrifice of any unnecessary tissue [11].

Thanks to these characteristics and to the technical refinements achieved in the last years, perforator propeller flaps find increasing indications in the reconstruction of different parts of the body.

### 3.1. Classification

A clear definition of propeller flap was given in 2009 by the advisory panel of the first Tokyo meeting on perforator and propeller flaps [12], who defined it as an “island flap that reaches the recipient site through an axial rotation.” The difference between a propeller flap and other pedicled flaps is that the rotation in the case of a propeller flap is “axial”: this means that the flaps turn around a pivot that is made of the pedicle and this is similar to a propeller.

According to the Gent consensus on perforator flaps [13] and to the advisory panel of the first Tokyo meeting on perforator and propeller flaps [12], perforator propeller flaps should be named after their nutrient vessels. They can be classified according to the type of nourishing pedicle.

- Subcutaneous pedicled propeller flap is based on a random subcutaneous pedicle and allows for rotations up to 90°.
- Perforator pedicled propeller flap is based on a skeletonized perforator pedicle. This is the most commonly used type of propeller flap and can be rotated up to 180°.
- Supercharged propeller flap is modification of the perforator pedicled propeller flap, in which a superficial or perforating vein of the flap is anastomosed to a recipient vein or an extra artery is anastomosed to a second arterial pedicle of the flap, to increase venous outflow or arterial inflow.

Recently, we described a novel type of propeller: the “axial propeller flap” [14] that includes propeller flaps based on known vessels (e.g., suprathrochlear artery and lingual artery) [14–16] and not on a perforator.

### 3.2. Surgical Technique

#### 3.2.1. Preoperative Planning

**Figure 1:**
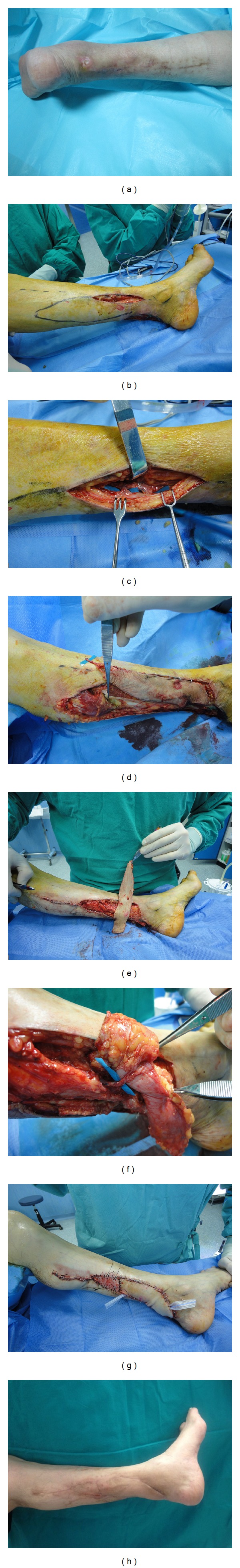
(a) Left Achilles tendon exposure after open repair. (b) The flap has been drawn around the perforator with the best sound. A wide exploratory incision is performed to visualize it. (c) Optimal perforator visualization, such as that shown in the picture, must be possible through the exploratory incision. When exposure is inadequate, the incision should be lengthened. In this case the proximal perforator was directed to the skin and the distal to the soleus muscle; plan B option for this case. (d) The flap has been islanded and left on the perforator alone to let the circulation settle. A superficial vein (greater saphenous vein in this case) should be preserved for venous supercharging whenever possible. A strip of soleus tendon is harvested for Achilles tendon reconstruction as described by Cavadas and Landin [10]. (e) The most convenient sense of rotation is chosen. While the flap's circulation settles before rotation, donor site closure can be accomplished. (f) After rotation, the pedicle is always double checked for torsions, traction, or kinking that must be, if present, immediately eliminated. (g) Closure must be obtained without any tension. Note that the flap is a little longer than required to compensate postoperative swelling. (h) Six months postoperative result shows complete flap survival.

A hand held Doppler probe is always sufficient for preoperative vascular assessment. The whole region should be investigated to have a clear preoperative picture of the location of all perforators or of the axial vessels. If a propeller axial flap is planned, identification of the vessel is easier because its position is constant and well known [14–16]. A flap is marked around the perforator with the best pulse and location (or around the axial vessel), possibly allowing for primary closure of the donor site (Figure 1(b)). Different options can be taken into account if more than one vessel is identified. An alternative flap is always planned as a “plan B.” An exploratory incision is planned without interfering with the alternative local flap(s) (Figure 1(c)) or such as to allow access to the recipient vessels, when plan B is a free flap. If possible, the skin incision is placed along previous scars or natural folds. A common mistake at the beginning is insufficiently wide exploratory incision that restrains vision and interferes with accurate identification of perforators. The incision is our window to the perforator and must be wide enough (Figure 1(c)).

#### 3.2.2. Flap Dissection

Through the exploratory incision suprafascial or subfascial dissection under loupe magnification is used to identify all the perforators around the defect. Once all perforators have been identified, the best one is chosen based on caliber, pulsatility, course and orientation, number and caliber of accompanying veins, and proximity to the defect and to a sensory nerve (the biggest perforators usually accompany sensory nerves all over the body). Then, in cases of suprafascial dissection, the fascial opening is widened and the perforator is freed from any surrounding tissue and dissected as long as possible (up to the source vessel) in order to achieve an adequate length of the pedicle along which to distribute the torsion [17]. Care should be taken in order to divide any attachment of the perforator to the surrounding tissues, like side branches (that must be ligated and not cauterized to avoid thermal damage to the perforator) or fibrous bands. As demonstrated by Wong et al. [18], the risk of vessel buckling is decreased when, for a perforator of 1 mm diameter, the vessel length is more than 3 cm. To prevent spasm, no tension must be placed on the perforator, which should be manipulated as little as possible.

If needed, the flap is redrawn around the chosen perforator. Differently from traditional flaps, traditional length/width ratios do not apply to propeller flaps because the pedicle usually penetrates the flap around its central part: this means that a 25/5 cm flap should not be considered a 5/1 flap but rather a 3/1 plus a 2/1 flap. The possibility of achieving donor-site closure should be the main concern about flap size, rather than concerns about flap perfusion [6, 19]. If perfusion is deemed insufficient, the flap should be supercharged, whenever possible. For this reason, adequate length of superficial veins (Figure 1(d)) and of other perforators entering the flap must be preserved.

#### 3.2.3. Flap Insetting

A crucial step in warranting survival of these flaps is to wait for the circulation to settle before flap rotation for at least 20 minutes (Figure 1(e)). After rotating the flap to its new position, its pedicle should be checked for twisting or buckling (Figure 1(f)) and further dissected if any limitation exists to an even distribution of the torsion. Clockwise and counterclockwise rotations are evaluated and the best one in terms of vessel rotation is chosen. The sense of rotation should be documented should the flap need to be reexplored. The flap is then secured in position and observed for color, capillary refilling, and bleeding (Figure 1(g)). If insufficient arterial inflow is observed due to arterial spasm caused by surgical manipulation, the flap should be brought back to its original position until spasm resolution (usually about 20 minutes) and its pedicle rinsed with lidocaine or papaverine. If the spasm persists after derotation, the flap should not be transferred anyway but rather left in place and delayed a few days before wound coverage [6].

#### 3.2.4. Postoperative Care

Limbs should be kept in a splint for the first postoperative days; compression on the flap should be avoided and elevation should be maintained for head and limbs flaps.

Flaps are checked every second hour during the first postoperative days, to allow for prompt identification of eventual complications.

### 3.3. Complications: Prevention and Management

#### 3.3.1. Arterial Insufficiency

This complication is extremely rare: accurate planning of the flap and choice of the perforator help preventing it. When, due to persistence of arterial spasm, the flap remains pale due to insufficient arterial inflow, the flap can be derotated to its original position for a few days before rotating it [4, 6].

#### 3.3.2. Venous Insufficiency

Venous congestion is the most frequent complication of propeller flaps, because veins are more prone to torsion than arteries. Venous insufficiency should be distinguished from the temporary congestion that often characterizes perforator flaps and fades out with stabilization of flow. True venous insufficiency worsens with time and should be promptly recognized and treated. When it is limited to an apical part of the flap, its evolution is observed. A small number of cases evolve in necrosis, which is usually superficial, so that deep vital tissue is still present at the recipient site.

Cases of mild venous congestions in thin flaps can be addressed with leech therapy.

When venous congestion is significant and worsens over time, reexploration and venous supercharging are the best option, in case a superficial or perforating vein of the flap was prepared during dissection. Should venous supercharging not be feasible, an alternative option is to temporarily derotate the flap (a few days) to relieve torsion on the pedicle [4, 6, 12] and let the circulation settle.

#### 3.3.3. Partial Necrosis

Total flap loss is rare. Partial necrosis seems to occur in about 5% of cases [6] and is often limited to the skin. After eschar removal, an adequate bed for a skin graft is often present. Healing by secondary intention is another alternative for small wounds.

### 3.4. Propeller Flaps in the Different Body Regions

#### 3.4.1. Head and Neck

The head and neck region is characterized by a very rich vascularization, and several local flaps are available for reconstruction. However, propeller perforator flaps allow turning a two-stage operation to a one-stage operation, thus simplifying reconstructions that usually require two or more procedures, accelerating recovery, and minimizing discomfort for the patient. Their freedom in design also allows for a better concealing of the scars.

Free-style flaps can be based on perforators of the facial artery and have been successfully used as propeller flaps for nasal ala reconstruction in a single stage [5–9, 11, 13, 20, 21].

One-stage nasal reconstruction with a perforator propeller flap from the forehead, named the suprathrochlear artery propeller perforator flap, has been reported as well [14, 15]. Other cutaneous, as well as mucosal intraoral, traditional flaps such as the lingual flaps [16] can be modified into a propeller flap based on their vascular pedicle for increased reconstructive possibilities.

The head and neck is the ideal donor site to start with when approaching propeller flap surgery for the following reasons:

- flaps in this area are more forgiving and have higher chances of survival compared, for example, to the limbs due to the rich vascular network of the head and neck;
- the head and neck vessels seem to better tolerate torsion and to suffer less from 180° rotations even if based on short pedicles;
- perforator vessels are usually very small and thus require that good skills are developed for their dissection.

#### 3.4.2. Lower Limb

In lower limb reconstruction, defects of the lower third of the leg are a challenging problem, due to the paucity of local tissues available for reconstruction [4]. Propeller flaps allow bringing proximal skin distally to cover average size defects that would otherwise require a free flap.

Free flaps are still the gold standard for large defects, but propeller perforator flaps are an appealing option for small and medium defects. Bajantri et al. [22] recommend their use for defects up to 50 cm^2^ in size; however, we believe that we are very far from establishing a maximum flap size, which depends on the patient's body and leg size, skin laxity, flap volume, perforator enrolled, adequate donor site closure and many other factors.

Based on the vascular supply of the lower limb, very long longitudinal flaps can be raised compared to other anatomical regions [6, 8]. Tibial posterior perforators seem to have an advantage over anterior tibial and peroneal artery perforators because they usually have a larger caliber and better veins [6].

When a deep defect has to be filled, a cuff of muscle can be transferred with the distal end of the perforator. The desired muscle is harvested around a perforator located in the tip of the flap, which is divided on a plane deeper to the muscle. The muscle cuff will be supplied by a reverse flow from the skin island through the divided perforator [4, 12]. This is an example of the degree of customization provided by these flaps. While we used to harvest musculocutaneous flaps in the past, to warrant vascular supply to the skin, now we moved to cutaneous-muscular flaps, where only the portion of muscle needed for the reconstruction (and not as a vehicle for the vessels) is taken with the flap. This optimizes outcomes at the recipient site and minimizes morbidity.

The donor defect can be closed primarily only in narrow longitudinal defects. Skin grafting of the donor site is often required. If a skin grafted donor site in the leg is considered unappealing, a free flap would avoid further scars in the leg [6]; when reconstruction with local flaps is planned, however, propeller flaps are often among the few available options and provide better aesthetic results compared to grafted adipofascial flaps.

Even when a free flap is needed, pedicled propeller flaps can be a valuable help for reconstruction. Cavadas and Teran-Saavedra [23] described a “razor flap,” in which a combination of the TDAP and the LD (Latissimus dorsi) flaps on the same thoracodorsal vessels permits a free rotation of the skin island and a great freedom of positioning. The flap was used for simultaneous release of popliteal retraction and circumferential resurfacing of the leg without increasing donor-site morbidity.

#### 3.4.3. Upper Limb

**Figure 2:**
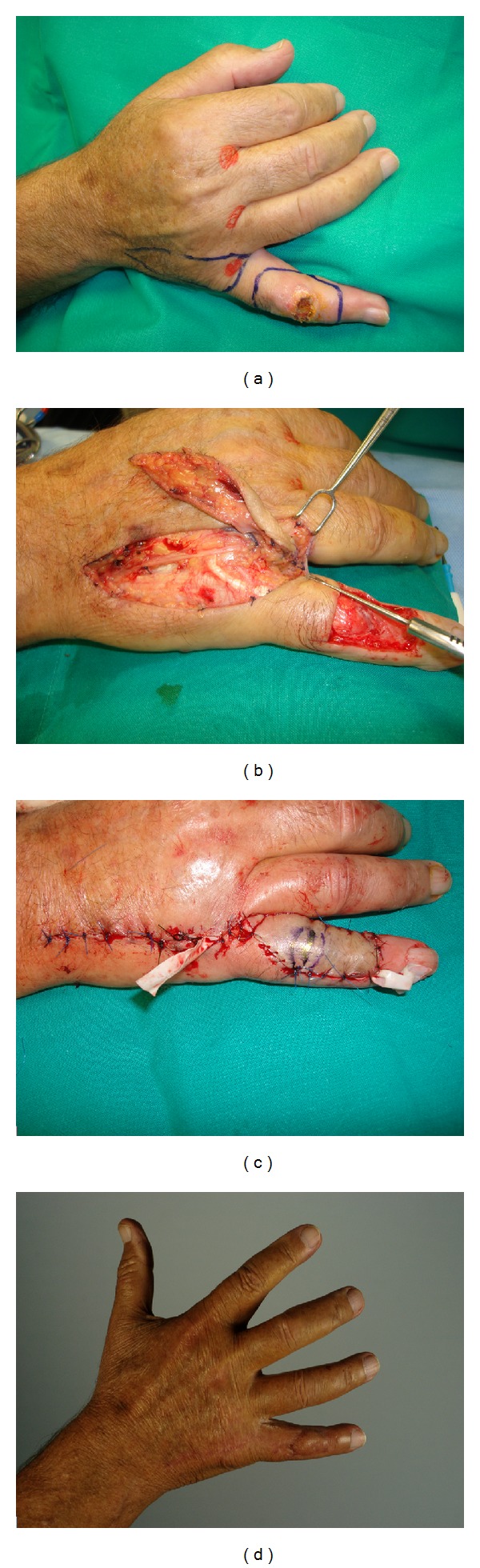
(a) Squamous cell carcinoma of the dorsal aspect of the little finger. Excision and reconstruction with a perforator propeller flap was planned. (b) Dissection view of the flap. (c) Immediate postoperative result. (d) Final result.

Clinical experiences with propeller flaps in upper limb are less numerous than in lower limbs, but with similar advantages. They allow reconstructing like-with-like, using a local and simple option, with the additional benefit of almost always allowing direct closure of the donor site. They have been used for reconstruction of the elbow and forearm region, and recently their use has been proposed for finger and hand defects [6, 9, 24–26] (Figure 2).

As for lower limb, propeller flaps also permit reconstructing defects of different tissue with a single flap. As an example, Battiston et al. [27] reported a composite teno-fasciocutaneous flap based on a perforator branch of the second dorsal intermetacarpal artery, for reconstruction of a complex defect of the dorsal aspect of the index finger.

A higher rate of venous insufficiency has been reported in the forearm. This is probably due to predominance of the superficial venous circulation, with subsequent venous engorgement of a flap based on the perforating veins alone. We advocate routine venous supercharging in the forearm to warrant sufficient venous drainage [6].

#### 3.4.4. Trunk

Although the potential of propeller flaps has been better documented in extremity surgery, their indications in reconstruction of trunk defects are steadily increasing.

Ang et al. [28] and Woo et al. [29] reported the use of propeller DIEP (deep inferior epigastric perforator) flaps rotated 180° for coverage of large abdominal defects, respectively, following resection of colorectal cancer cutaneous metastasis and fibrodermatosarcoma protuberans. A preexpanded propeller DIEP flap has been reported by Cheng and Saint-Cyr [30] for reconstruction of an abdominal burn scar.

Other well-known perforator flaps are routinely used as pedicled propeller flaps for trunk reconstruction. Their long pedicle allows for a great range of freedom, allowing for an extensive arc of rotation, and makes these flaps particularly suitable for being rotated with little concerns on vessels' torsion and blood supply.

Several authors reported reconstruction of complex abdominal or pelvic defects with pedicled propeller ALT (anterolateral thigh) flaps [23, 31–33].

ALT, SGAP (superior gluteal artery perforator) flap, IGAP (inferior gluteal artery perforator) flap, and TDAP (toracodorsal artery perforator) flap have been described as pedicled propeller flaps for reconstruction of difficult wounds of axillary, gluteal, or inguinal regions following resection of hidradenitis suppurativa [6, 34–37].

ICAP (intercostal artery perforator) flaps, LTAP (lateral thoracic artery perforator) flaps, and TDAP flaps are a valuable option for partial breast reconstruction [7, 38–40]; LTAP, ICAP, and IMAP flaps find indication in reconstruction of other complex thoracic defects [38, 41, 42].

## 4. Conclusions

Perforator propeller flaps are a valid reconstructive option for difficult wounds and can be raised from any part of the body. Their harvesting is easy and fast, provided that an accurate dissection technique is applied, and allows for great freedom in design and choice of the donor site.

Propeller perforator flaps represent an alternative to free flaps when traditional flaps are not an option, allow to reconstruct even complex wounds with local tissues and a low donor-site morbidity, and present several advantages over traditional pedicled flaps: their freedom in design allows to reconstruct complex defects usually requiring multiple procedures in a single stage, accelerating recovery, minimizing morbidity and discomfort for the patient, and allowing a better aesthetic result and concealing of scars.
